# Cross-Linking Mass Spectrometry Uncovers Interactions Between High-Density Lipoproteins and the SARS-CoV-2 Spike Glycoprotein

**DOI:** 10.1016/j.mcpro.2023.100600

**Published:** 2023-06-19

**Authors:** Sean A. Burnap, Ana Maria Ortega-Prieto, Jose M. Jimenez-Guardeño, Hashim Ali, Kaloyan Takov, Matthew Fish, Manu Shankar-Hari, Mauro Giacca, Michael H. Malim, Manuel Mayr

**Affiliations:** 1 Department of Chemistry, Physical and Theoretical Chemistry Laboratory, University of Oxford, Oxford, UK; 2 The Kavli Institute for Nanoscience Discovery, Dorothy Crowfoot Hodgkin Building, University of Oxford, Oxford, UK; 3 King's College London British Heart Foundation Centre, School of Cardiovascular Medicine and Sciences, London, UK; 4 Department of Infectious Diseases, School of Immunology and Microbial Sciences, King's College London, London, UK; 5 Division of Virology, Department of Pathology, Addenbrooke's Hospital, University of Cambridge, Cambridge, UK; 6 Department of Intensive Care Medicine, Guy's and St Thomas' NHS Foundation Trust, London, UK; 7 Centre for Inflammation Research, Institute of Regeneration and Repair, University of Edinburgh, Edinburgh, UK; 8 Royal Infirmary of Edinburgh, NHS Lothian, Edinburgh, UK

**Keywords:** XL-MS, HDL, SARS-CoV-2, ApoD, spike

## Abstract

High-density lipoprotein (HDL) levels are reduced in patients with coronavirus disease 2019 (COVID-19), and the extent of this reduction is associated with poor clinical outcomes. While lipoproteins are known to play a key role during the life cycle of the hepatitis C virus, their influence on coronavirus (CoV) infections is poorly understood. In this study, we utilize cross-linking mass spectrometry (XL-MS) to determine circulating protein interactors of the severe acute respiratory syndrome (SARS)-CoV-2 spike glycoprotein. XL-MS of plasma isolated from patients with COVID-19 uncovered HDL protein interaction networks, dominated by acute-phase serum amyloid proteins, whereby serum amyloid A2 was shown to bind to apolipoprotein (Apo) D. XL-MS on isolated HDL confirmed ApoD to interact with SARS-CoV-2 spike but not SARS-CoV-1 spike. Other direct interactions of SARS-CoV-2 spike upon HDL included ApoA1 and ApoC3. The interaction between ApoD and spike was further validated in cells using immunoprecipitation-MS, which uncovered a novel interaction between both ApoD and spike with membrane-associated progesterone receptor component 1. Mechanistically, XL-MS coupled with data-driven structural modeling determined that ApoD may interact within the receptor-binding domain of the spike. However, ApoD overexpression in multiple cell-based assays had no effect upon viral replication or infectivity. Thus, SARS-CoV-2 spike can bind to apolipoproteins on HDL, but these interactions do not appear to alter infectivity.

## Highlights

• COVID-19 plasma XL-MS uncovered acute-phase protein interaction networks.
• XL-MS upon SARS-CoV-2 spike and HDL identified multiple spike interactors.
• Affinity purification-MS confirmed an interaction between spike and ApoD.
• Structural modelling determined ApoD to interact with the receptor-binding domain.
• ApoD overexpression failed to influence SARS-CoV-2 replication and infection.

## Graphical Abstract

**Figure:**
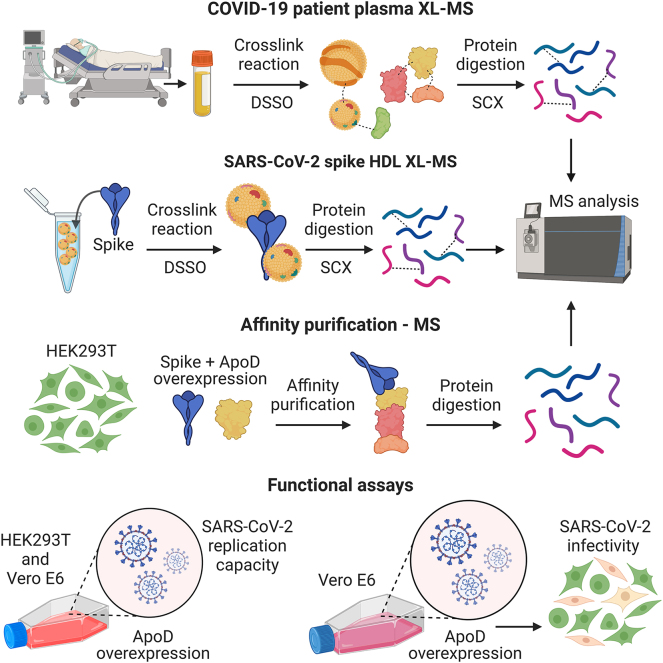

## In Brief

Here we utilized cross-linking mass spectrometry (XL-MS) to investigate circulating protein interaction networks in the context of SARS-CoV-2. The spike glycoprotein was determined to interact with multiple proteins on high-density lipoprotein including ApoD. The interaction between spike and ApoD was confirmed in cells through affinity purification-MS. XL-MS upon recombinant spike and ApoD, coupled with structural modeling, identified ApoD to interact within the spike receptor-binding domain. However, ApoD overexpression in cultured cell lines did not influence SARS-CoV-2 replication or infection.

High-density lipoprotein (HDL) levels are inversely associated with disease severity and poor clinical outcomes in patients with coronavirus disease 2019 (COVID-19) (1, 2, 3, 4, 5, 6). Importantly, it was recently shown that individuals with high antecedent HDL levels were at a lower risk of severe acute respiratory syndrome (SARS) coronavirus (CoV) 2 infection (7). Similarly, data from the UK biobank have associated increased HDL-cholesterol and apolipoprotein (Apo)-A1 levels with decreased risk of SARS-CoV-2 infection (8). However, Mendelian randomization analyses did not yield evidence for genetically increased HDL-cholesterol levels being causally associated with SARS-CoV-2-related outcomes (8). Thus, while high HDL levels lower the risk of severe SARS-CoV-2 infection, mechanistic insights regarding a potential causal involvement of HDL are lacking (9).

HDL has been shown to stimulate the cellular uptake of SARS-CoV-2 spike protein and promote infection, with the mechanism being deemed to be through cholesterol binding rather than protein–protein interaction (10, 11). Conflicting data, however, suggest HDL, at high concentrations, may also be able to reduce the cytopathic effect of SARS-CoV-2 infection, posing a dose dependency in the functional consequence of HDL upon SARS-CoV-2 (12). Additionally, the capability of HDL to act with an antiviral capacity was related to the antioxidant potential of HDL, suggesting HDL protein content to be important (12).

Cross-linking mass spectrometry (XL-MS) is widely used in the determination of protein–protein interaction networks at a proteome-wide scale (13, 14). XL-MS data can be further leveraged through distance constraints and integrative protein modeling to understand modes of interaction (13, 15). HDL is the most protein-rich lipoprotein particle. Its protein composition is highly heterogenous, but studying HDL has relied upon low-resolution methodologies thus far. The mechanisms by which protein composition and protein interactions are regulated on HDL are largely unexplored. We have previously used XL-MS to uncover a direct interaction between proprotein convertase subtilisin/kexin type 9 (PCSK9) and ApoA1 on HDL (16). The application of XL-MS to HDL (16, 17) in the context of SARS-CoV-2 could provide molecular insights regarding the immune-modulating properties of HDL.

In the present study, we used XL-MS on plasma from patients with COVID-19 admitted to intensive care to determine acute-phase HDL interaction networks. Moreover, we utilized XL-MS to determine interactions between the SARS-CoV-2 spike protein and HDL. An interaction between spike and ApoD on HDL was confirmed using affinity purification-MS as well as XL-MS upon recombinant ApoD and spike. Integrative structural modeling mapped the ApoD interaction to the receptor-binding domain of the spike. However, overexpression of ApoD in cell-based assays did not alter SARS-CoV-2 replication and infection.

## Experimental Procedures

### COVID-19 Clinical Cohort

We obtained plasma samples from patients with severe disease (*i.e.*, WHO ordinal scale > 5, n = 16) within 6 days of admission to the ICU of King’s College Hospital, London, UK (1, 18). Plasma was collected in EDTA BD Vacutainer tubes (BD, 362,799) and centrifuged at 2000*g* for 15 min. Recruitment of patients occurred between March 2020 and July 2020. The study was conducted in accordance with the Declaration of Helsinki and approved by an institutional review board (REC19/NW/0750). Written informed consent was obtained directly from patients (if mentally competent) or from the next of kin or professional consultee. The consent procedure was then completed with retrospective consent if the patient regained capacity. Plasma samples were depleted using the High Select Top14 Abundant Protein Depletion spin columns (Thermo Fisher).

### Cross-Linking MS

Protein–protein interactions within HDL, plasma, and recombinant proteins were interrogated using the MS-cleavable cross-linker disuccinimidyl sulfoxide (DSSO, Thermo Fisher) and DMTMM (Merck), following recently published protocols (19, 20). Recombinant SARS-CoV-1 and SARS-CoV-2 spike protein (R&D 10683-CV-100 and Thermo Fisher RP-87680, respectively) was mixed with HDL isolated by potassium bromide (kBr) density ultracentrifugation (Merck, LP3-5MG) diluted in PBS (pH 7.5) at a concentration of 1 μg/μl and allowed to incubate overnight at 4 °C. DSSO was prepared as a 50 mM stock solution in DMSO and added to protein mixtures at a final concentration of 1 mM for 30 min at room temperature. DMTMM was prepared in PBS and used at a final concentration of 10 mM. Cross-linking reactions were quenched through the addition of Tris-HCL (pH 8.5) at a final concentration of 50 mM for 30 min at room temperature. Cross-linked protein samples were then digested and purified as described below prior to fractionation using a strong-cation exchange (SCX) Dionex UltiMate 3000 RSLC system (Luna 5 μm, 100 Å, 50 × 2 mm. Phenomenex). The resulting peptide fractions were dried using a speed vac (Thermo Fisher, Savant SPD131DDA) and desalted robotically using C18 cartridges (Bravo AssayMAP, Agilent Technologies); peptides were then resuspended in 5% DMSO/10% FA/85% H_2_O prior to MS-analysis. Experiments using recombinants proteins (ApoD, a.a 21-189, Abcam ab184598, Full-length Spike a.a 11-1208, Thermo Fisher RP-87680 and Spike-RBD, a.a 319-541, R&D 10500-CV) only involved mixing at an equimolar ratio prior to overnight incubation at 4 °C and following the same protocol as described earlier. Experiments involving depleted plasma followed the same XL-MS protocol above.

### In-Solution Protein Digestion

Protein samples were denatured by the addition of a final concentration of 6 M urea and 2 M thiourea and reduced by the addition of a final concentration of 10 mM dithiothreitol (DTT) followed by incubation at 37 °C for 1 h, 240 rpm. The samples were then cooled down to room temperature before being alkylated by the addition of a final concentration of 50 mM iodoacetamide followed by incubation in the dark for 30 min. Pre-chilled (−20 °C) acetone (10× volume) was used to precipitate the samples overnight at −20 °C. Samples were centrifuged at 14,000*g* for 40 min at 4 °C, and the supernatant was subsequently discarded. Protein pellets were dried using a speed vac, resuspended in 0.1 M TEAB buffer, pH 8.0, containing 0.02% ProteaseMax surfactant (in the case for lipoprotein samples) and MS grade Trypsin/Lys-C (Promega Cooperation) (1:25 enzyme: protein) and digested overnight at 37 °C, 240 rpm. Digestion was stopped by acidification with TFA. Peptides were then purified robotically, using C18 cartridges (Bravo AssayMAP, Agilent Technologies).

### LC-MS/MS Analysis

Dried peptide samples for label-free analyses were reconstituted with 0.05% TFA in 2% ACN and separated by a nanoflow LC system (Dionex UltiMate 3000 RSLC nano). Samples were injected into a nano-trap column (Acclaim PepMap100 C18 Trap, 5 mm × 300 μm, 5 μm, 100 Å), at a flow rate of 25 μl/min for 3 min, using 0.1% FA in H_2_O. The following nano-LC gradient was then run at 0.25 μl/min to separate the peptides: 0 to 10 min, 4 to 10% B; 10 to 75 min, 10 to 30% B; 75 to 80 min, 30 to 40% B; 80 to 85 min, 40 to 99% B; 85 to 89.8 min, 99% B; 89.8 to 90 min, 99 to 4% B; 90 to 120 min, 4% B; where A = 0.1% FA in H_2_O, and B = 80% ACN, 0.1% FA in H_2_O. The nano column (EASY-Spray PepMap RSLC C18, 2 μm 100 Å, 75 μm x 50 cm), set at 40 °C was connected to an EASY-Spray ion source (Thermo Scientific). Spectra were collected from an Orbitrap mass analyzer (Orbitrap Fusion Lumos Tribrid, Thermo Scientific) using full MS mode (resolution of 120,000 at 400 m/z) over the mass-to-charge (m/z) range 375 to 1500. Data-dependent MS2 scan was performed using Quadrupole isolation in Top Speed mode using CID activation and ion trap detection in each full MS scan with dynamic exclusion enabled.

### LC-MS/MS Acquisition Strategy for Cross-Linked Peptides

Cross-linked peptide samples resuspended in 5% DMSO/10% FA/85% H_2_O were separated by a nanoflow LC system (Dionex UltiMate 3000 RSLC nano), utilizing the same gradient as above. The nano column (EASY-Spray PepMap RSLC C18, 2 μm 100 Å, 75 μm × 50 cm), set at 40 °C, was connected to an EASY-Spray ion source (Thermo Scientific). Spectra were collected from an Orbitrap mass analyzer (Orbitrap Fusion Lumos Tribrid, Thermo Scientific) using full MS mode (resolution of 60,000 at 400 m/z) over the mass-to-charge (m/z) range 375 to 1600, utilizing an XLMS cleavable MS2-MS3 method in the case of DSSO, or an MS2 only method for DMTMM. Peptides of charge 3 to 8 were chosen and sorted, favoring the highest charged state. Data-dependent MS2 scan was performed using Quadrupole isolation with a cycle time of 5 s with CID activation and Orbitrap detection at 30,000. A targeted mass difference of Delta M1: 31.9721 was defined to detect the presence of DSSO cross-linked peptides for their analysis in an MS3 scan. Data-dependent MS3 scans were performed using Quadrupole isolation with CID activation and ion trap detection.

### MS Database Search and Analysis

Thermo Scientific Proteome Discoverer software (version 2.2.0.388) was used to search non-cross-linked raw data files against the human database (UniProtKB/Swiss-Prot version 2018_02, 20,400 protein entries) using Mascot (version 2.6.0, Matrix Science). The mass tolerance was set at 10 ppm for precursor ions and 0.8 Da for fragment ions. Trypsin was used as the digestion enzyme with up to two missed cleavages being allowed. Carbamidomethylation of cysteines and oxidation of methionine residues were chosen as fixed and variable modifications, respectively. Label-free quantification was conducted through the in-built Minora feature detection node, alongside retention time alignment using the feature mapper node, with a maximum retention time shift of 10 min being allowed. Unique and Razor peptide precursor areas were used for quantification, and cross-sample normalization was achieved using the total peptide amount. MS/MS-based peptide and protein identifications were validated with the following filters, a peptide probability of >95.0% (as specified by the peptide prophet algorithm), a protein probability of >99.0%, and at least two unique peptides per protein.

DSSO XLMS acquired data was searched in Thermo Scientific Proteome Discoverer software (version 2.5) using the third-party XlinkX node developed by the Heck lab (21). The inbuilt MS-cleavable MS2/MS3 DSSO search strategy was used. XL-MS raw files for plasma analyses were searched against a curated human database of previous in-house DDA analyses (1048 protein entries), while HDL XLMS raw files were searched against an in-house HDL proteome database containing sequence information for 348 proteins including the SARS-CoV-2 spike protein. Sequest HT was used with Trypsin as the digestion enzyme with up to two missed cleavages being allowed. Methionine oxidation, DSSO modifications of lysine residues, and N-terminal acetylation were set as dynamic modifications, and carbamidomethylation of cysteines was set as static. MS2_MS3 strategy was set, and the DSSO/+158.004 Da (K) cross-link modification was used while keeping all other settings default. DMTMM XLMS data were searched using pLink2 (22), peptide mass accuracy was ±10 ppm, fragment mass accuracy was ±20 ppm, and a 5% FDR filter was applied at the spectral level. XLMS data visualization was conducted using xiview.org (23).

### Lipoprotein Immuno-Depletion

HDL was immuno-isolated from healthy volunteer plasma (n = 8) using human HDL-specific IgY affinity columns according to the manufacturer’s instructions (Genway Biotech). Briefly, 40 μl of plasma was diluted 10-fold in 360 μl TBS buffer (10 mM Tris, 150 mM NaCl, pH 7.4). Diluted plasma was then added to TBS equilibrated antibody beads and incubated at room temperature with end-over-end rotation for 15 min. Flow through, HDL-depleted plasma was then collected through centrifugation at 500*g*. The removal of non-specifically bound proteins from the antibody beads was achieved using 500 μl of wash buffer (TBS, 0.05% Tween-20) a total of three times. HDL was then stripped from the antibody beads by the addition of 500 μl stripping buffer (0.1 M Glycine, pH 2.5), twice. The antibody columns were then regenerated using a series of stripping buffer wash steps, followed by the addition of neutralization buffer (100 mM Tris-HCI, pH 8.0), and finally the resuspension in 500 μl TBS containing 0.02% sodium azide for storage. HDL-depleted plasma was stored at −80 °C until further processing.

The same plasma samples were depleted of ApoB-containing lipoproteins through the use of a LipoSep IP immunoprecipitation kit (Sun Diagnostics) according to the manufacturer’s instructions. In brief, equal parts plasma and IP reagent were mixed and incubated at room temperature for 10 min prior to centrifugation at 10,000*g* for 10 min. Resulting plasma supernatants depleted of ApoB-containing lipoproteins were stored at −80 °C until further processing.

### Cell Culture

HEK293T cells (ATCC CRL-3216) were cultured in Dulbecco’s modified Eagle medium (DMEM) with 1 g/l glucose (Life Technologies). Vero E6 cells (kindly provided by W. Barclay (Imperial College London)) and HEK293T-ACE2 (24) cells were cultured in complete DMEM GlutaMAX (Gibco). All cell lines were supplemented with 10% fetal bovine serum (FBS) (Life Technologies) plus a final concentration of 100 IU/ml penicillin and 100 μg/ml streptomycin, or without antibiotics when required for transfections. Cells were incubated at 37 °C, 5% CO_2_. HEK293T-ACE2 cells constitutively expressing human ACE2 were generated by stable transduction of HEK293T cells with a pMIGR1-blast-ACE2 (25) vector and maintained with 10 μg/ml blasticidin selection.

### Plasmid Transfection and Cell Lysis

Human ApoD and PGRMC1 encoding plasmids were obtained from Origene (pCMV6-ApoD: RC200503, pCMV6-PGRMC1: RC201918). pAAV-CMV-GFP was obtained from L. Zentilin (Molecular Medicine Lab, International Centre for Genetic Engineering and Biotechnology, Trieste, Italy). The SARS-CoV-2 spike coding sequence (NCBI accession number NC_045512.2) was codon-optimized and synthesized with a V5 tag at the C-terminus and then cloned into the pZac 2.1 AAV (pAAV-Spike) vector under the control of a CMV promoter. The untagged SARS-CoV spike plasmid was a gift from the W. Barclay laboratory. Plasmid DNA was mixed with the FuGENE HD transfection reagent (Promega) in Opti-MEM (Thermo Fisher) at a ratio of 3:1 (FuGENE:DNA) and complexes were allowed to form for 15 min. DNA complexes were then added to cells culture to 70% confluency in 6-well plates or T25 flasks in a slow drop-wise manner. Between 24 to 48 h post-transfected cells were lysed by sonication in IP buffer (20 mM HEPES, 150 mM NaCl, 1.5 mM MgCl_2_, 0.5 mM DTT, 0.4% NP-40, pH 7.8) with complete EDTA-free protease-inhibitor (Roche).

### Antibodies

Antibodies against the following targets were used: V5-tag (R960-25), myc-tag (MA1-980), SARS-CoV-1 spike (MA1-41093), SARS-CoV-2 nucleocapsid (Sino Biological 40143-R001) and ApoD (ab108191).

### Immunoprecipitation

Protein G Dynabeads for immunoprecipitation (Thermo Fisher) were used in this study. Briefly, antibodies were added to cell lysates (100 μg) and allowed to incubate overnight at 4 °C with end-over-end mixing. Pre-washed Dynabeads (50 μl) were then added to lysate/antibody mixtures with 1 h incubation at room temperature with end-over-end mixing. Antibody–Protein G complexes were subsequently washed three times with PBST (0.05 % Tween-20) and eluted in glycine buffer (0.1 M glycine, pH 2.5). Eluted protein mixtures were then neutralized with 1 M Tris-HCL pH 8.0.

### Immunoblotting

Laemmli sample buffer (4×) (62.5 mM Tris-HCL, pH 6.8, 10% glycerol, 1% SDS, 0.005% bromophenol blue and 10% 2-mercaptoethanol) or without 2-mercaptoethanol was mixed with protein samples and boiled at 95 °C for 10 min. Protein samples were separated using either 4 to 12% Bis-Tris or 3 to 8% Tris-Acetate gradient gels (Thermo scientific) in MOPS and Tris-Acetate SDS running buffer, respectively (Thermo Scientific), at 130V for 90 min. Gels were either stained for total protein using SimplyBlue Safe Stain (Thermo Fisher) or proteins were transferred onto nitrocellulose membranes in ice-cold transfer buffer (25 mM Tris-base pH 8.3; 192 mM glycine; 20% methanol) at 350 mA for 2 h. Ponceau S red staining was used to determine efficient transfer and equal loading before membranes were blocked in 5% fat-free milk powder in PBS containing 0.1% Tween-20 (PBST) (Sigma). Membranes were incubated in primary antibodies made to appropriate concentrations in 5% bovine serum albumin (BSA) in PBST overnight at 4 °C. The membranes were then incubated in the appropriate light-chain specific peroxidase-conjugated secondary antibody in 5% milk/PBST. Membranes were then washed for three times in PBST for 15 min. Western blots were developed using enhanced chemiluminescence (GE Healthcare) on photographic films (GE Healthcare).

### XL-MS Driven Interaction Modelling

Cross-links obtained from recombinant protein XL-MS experiments were used as distance constraints to drive the modeling of the interaction between ApoD and spike. Both the DisVis web server (DisVis Webserver (uu.nl)) (26, 27) and the HADDOCK docking tool (HADDOCK Web Server (uu.nl)) (15, 28, 29) were used. AlphaFold2 was also utilized to predict the structure of the ApoD and SARS-CoV-2 RBD complex. AlphaFold2 modeling was achieved through ColabFold v1.5.2, keeping all options default (30).

### SARS-CoV-2 Replication and Infection Cell Assays

SARS-CoV-2 Strain England 2 (England 02/2020/407073) was obtained from Public Health England. Viral stocks were produced by infecting Vero E6 cells using an MOI of 0.01. Virus-containing supernatants were collected 72 h after infection and centrifuged at 1500 rpm for 10 min, aliquoted, and stored at −80 °C. The infectious virus titers were determined by plaque assay in Vero E6 cells.

HEK293T-ACE2 and Vero E6 cells transfected with the indicated plasmids (pCMV6-ApoD and empty pCMV6 vector) were infected 48 h after transfection with SARS-CoV-2 at the indicated MOIs for 1 h. Cells were then washed with PBS and cultured in fresh medium for a further 48 h. Virus production in the culture supernatants was quantified by plaque assay using Vero E6 cells.

### Immunofluorescence

Vero E6 cells transfected with the indicated plasmids and infected with SARS-CoV-2 were fixed at indicated times with 4% PFA for 15 min at room temperature. Then, cells were washed twice with PBS and permeabilized with 0.1% Triton X-100 (Sigma-Aldrich 1086431000) for 15 min at room temperature with gentle shaking. Later, cells were washed twice with PBS and blocked with PBS containing 10% FBS for 1 h at room temperature. After blocking, the supernatant was removed and the samples were incubated with a SARS-CoV-2 nucleoprotein-specific antibody (dilution 1:250, Sino Biological, 40143-R001) overnight at 4 °C in PBS containing 10% FBS. Cells were then washed twice in PBS and a secondary antibody (dilution 1:500, Invitrogen, Ref A21206) was added and incubated for 1 h in PBS containing 10% FBS. Cells were then washed twice in PBS and nuclei were stained using DAPI (dilution 1∶10,000, Sigma, MBD0015-1Ml). Later, cells were examined by using an AID reader system according to the manufacturer’s instructions.

### Experimental Design and Statistical Rationale

To determine protein–protein interaction networks by XL-MS, experiments were conducted on pooled plasma and HDL samples. XL-MS was conducted upon pooled plasma from patients with COVID-19 from six biological replicates. HDL interactomics was conducted on commercially available pooled HDL from healthy donors (Merck, LP3-5MG) with and without supplementation of the SARS-CoV-1 and SARS-CoV-2 spike protein. Apolipoprotein distribution on lipoprotein particles through depletion and MS analysis was conducted upon eight biological replicates of healthy donor plasma with a matched design, whereby plasma samples from the same individuals were either depleted or non-depleted of lipoprotein. Affinity purification MS experiments were conducted with a minimum of three biological replicates per condition, that is, a different expression per sample. Significance was determined by the Welch’s *t* test. The ability of ApoD to modulate SARS-CoV-2 replication was conducted upon four biological replicates for each plasmid condition in 293T-ACE2 expressing cells, while two biological replicates per MOI were used in Vero E6 cells.

## Results

### XL-MS of Plasma from Patients With COVID-19

**Fig. 1:**
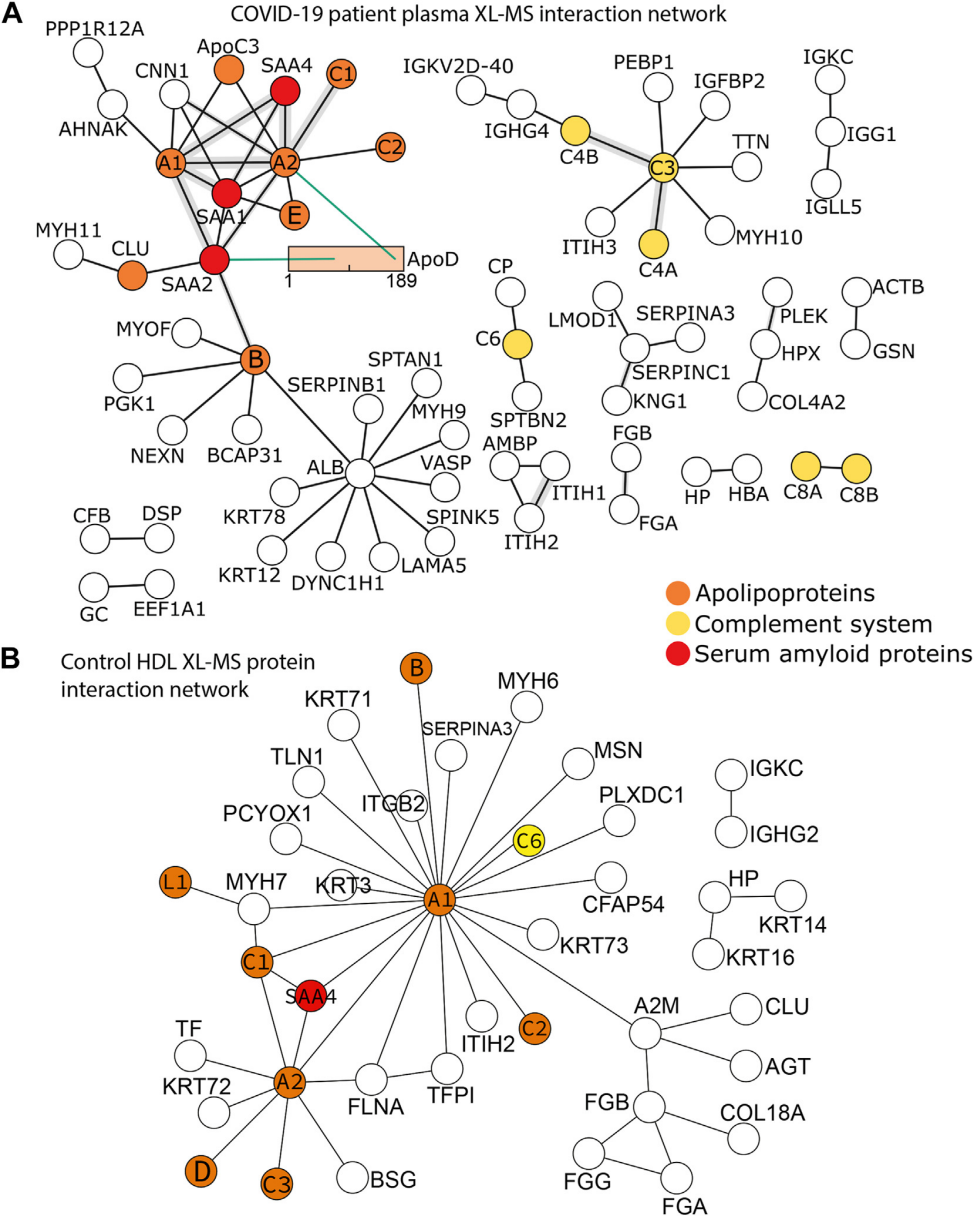
**XL-MS upon plasma derived from patients with COVID-19.***A*, plasma isolated from patients in intensive care with confirmed COVID-19 (n = 6) was pooled and crosslinked using DSSO. The subsequent interprotein crosslinks identified by XL-MS are represented as an interaction network, 125 crosslinks across 68 proteins. Crosslinks had a minimum of two associated crosslink spectral matches (CSMs) and an XlinkX score of >40. The amino acid sequence of ApoD has been expanded, highlighting different regions of protein interaction. Apolipoproteins are in orange, serum amyloid proteins (SAA) are in *red*, and complement factors are in *yellow*. *B*, HDL interactions in healthy controls were determined following the same XL-MS workflow and the resulting inter-protein crosslinks identified are visualised as an interaction network. XL-MS data visualisation was conducted in xiview.org. All protein abbreviations are gene names as determined by Uniprot.

We conducted XL-MS of pooled plasma from patients with COVID-19 in intensive care (n = 6) (1). To the best of our knowledge, XL-MS on such a complex matrix as plasma has yet to be attempted in COVID-19. The cross-linked plasma pool underwent proteolytic digestion followed by SCX-based peptide fractionation, enriching for cross-linked peptides. The greatest hindrance in the analysis of plasma lies in the large dynamic range of protein concentrations, a feature we partially overcome in this study through the depletion of abundant proteins (see albumin cluster, Fig. 1*A*). Identified cross-links were first filtered to have at least two cross-link spectral matches (CSMs) per cross-link and interprotein cross-links identified are displayed as a protein interaction network (125 cross-links across 68 proteins, Fig. 1*A*). As expected, prominent interaction clusters in our XL-MS analysis contained multiple members of the complement family, with complement C3 acting as a central hub for complement C4a and C4b, as well as interactions between known regulators of coagulation such as kininogen-1 (KNG1) and antithrombin-III (SERPINC1, Fig. 1*A*).

Another core protein interaction cluster observed centered around known HDL components, including the core apolipoproteins ApoA1 and ApoA2 (Fig. 1*A* and supplemental Table S1). The acute-phase proteins serum amyloid A1 (SAA1) and A2 (SAA2) interacted with multiple HDL-associated apolipoproteins, including ApoD, which we have previously observed to interact with SARS-CoV-2 spike using immunoprecipitation-MS in plasma from patients with COVID-19 (1) (Fig. 1*A*). SAA1 and SAA2 both act as exchangeable apolipoproteins during inflammation and remodel and displace proteins upon HDL (31). ApoD was seen to interact with ApoA2 and SAA2. Interestingly, the C-terminal region of ApoD was observed to be cross-linked with ApoA2, while SAA2 interacted within the central region of ApoD (Fig. 1*A*). SAA2 was also observed to directly interact with ApoB and could therefore act as a bridge in the potential interaction between spike and other lipoproteins apart from HDL (Fig. 1*A*). Central to this observation is the known ability of serum amyloid proteins to exchange between lipoprotein species (31).

The interactions between ApoA1 and SAA proteins were also confirmed by XL-MS in pooled COVID-19 plasma without depletion of abundant proteins (173 cross-links across 117 proteins, supplemental Fig. S1 and supplemental Table S2). As expected, protein interactions involving albumin dominated in non-depleted plasma (63 cross-links across 49 proteins). A distinct interaction cluster involving HDL and SAA proteins, however, could still be observed given the high levels of SAA1 and SAA2 in the circulation of patients with COVID-19 (1).

To further explore protein remodeling that could be specifically occurring on HDL as a consequence of the acute inflammatory setting of COVID-19, HDL from healthy controls was isolated by ultracentrifugation and analyzed by XL-MS to determine the baseline HDL interactome (Fig. 1*B* and supplemental Table S3). ApoD was again found to cross-link with ApoA2, while no interactions between ApoA1 and SAA1 or SAA2 were observed, revealing SAA1 and SAA2 to interact with ApoA1 during acute inflammation (Fig. 1*B*).

### ApoD is Predominantly HDL-Bound

**Fig. 2:**
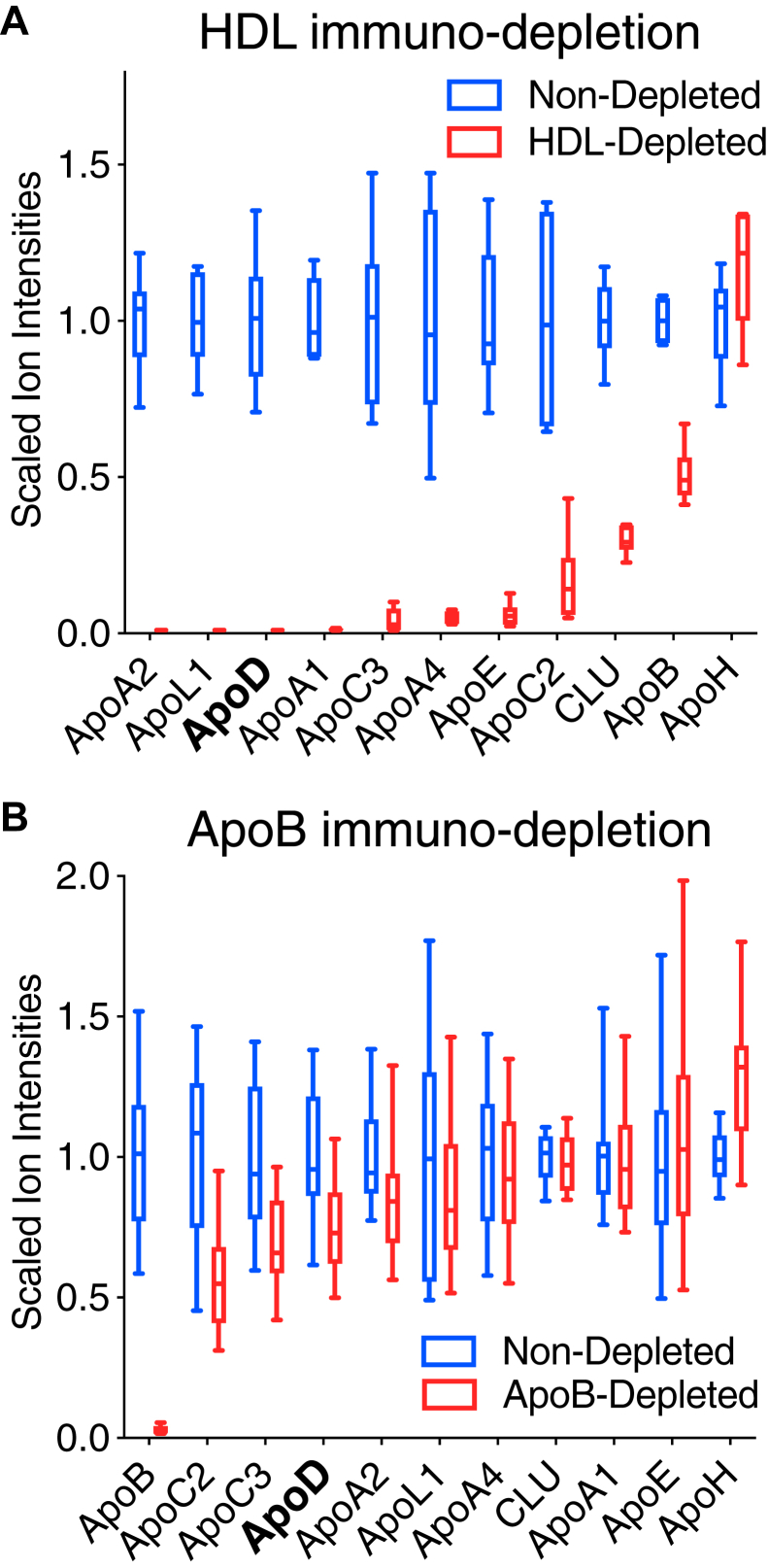
**Lipoprotein distribution of ApoD.***A*, fasted plasma was depleted of HDL utilizing immuno-depletion methods and apolipoprotein profiles were quantified by DDA-based label-free quantitation upon non-depleted and depleted samples (n = 8 healthy individuals). *B*, the same MS measurements were performed after the depletion of ApoB-containing lipoproteins.

While ApoD is thought to be primarily carried by HDL (32, 33, 34), it has previously been implicated in mediating the direct interaction between HDL and LDL (35). To confirm the proportion of ApoD that exists in a free or HDL-bound state, we depleted lipoproteins utilizing immuno-depletion approaches. The apolipoprotein profiles of non-depleted and lipoprotein-depleted plasma were determined by DDA-based label-free quantitation. ApoD was absent from plasma upon the removal of HDL, as confirmed by the efficient depletion of both ApoA1 and ApoA2 (Fig. 2*A*). However, the removal of a proportion of ApoB upon HDL depletion could lead to an overestimation in the level of circulating ApoD that is associated with HDL (Fig. 2*A*). Therefore, we depleted plasma of ApoB-containing lipoproteins using another immunoprecipitation method for ApoB and demonstrated that only a small amount of ApoD is carried by LDL or VLDL (Fig. 2*B*).

### XL-MS to Map Protein Interactions of SARS-CoV-2 Spike With HDL

**Fig. 3:**
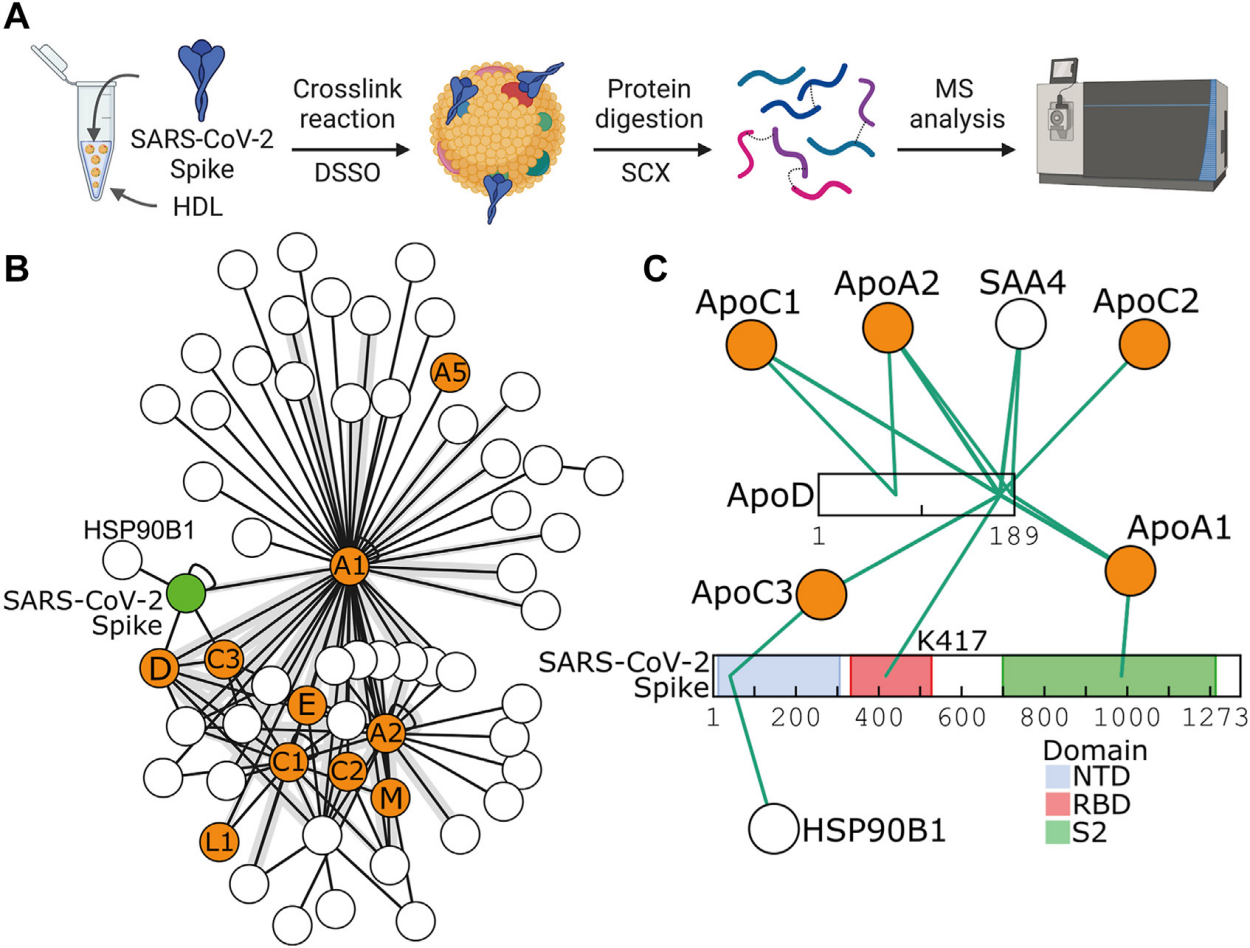
**XL-MS upon isolated HDL and SARS-CoV-2 spike protein.***A*, a schematic representation of the XL-MS workflow conducted upon mixtures of ultracentrifuge-isolated HDL and spike protein. *B*, inter-protein crosslinks identified within isolated HDL supplemented with spike protein are represented as an interaction network, 404 crosslinks across 60 proteins. Crosslinks had a minimum of two associated crosslink spectral matches (CSMs) and an XlinkX score of >10. *C*, protein-protein interactions identified by XL-MS between ApoD and spike are separately visualized. Spike structural domains are coloured; NTD: *blue*, RBD: *red* and S2: *green*. XL-MS data visualization was conducted in xiview.org. All protein abbreviations are gene names as determined by Uniprot.

Next, we used XL-MS upon human HDL to determine whether the SARS-CoV-2 spike can interact with apolipoproteins (Fig. 3*A*). Recombinant spike protein was added to pooled isolated HDL prior to the addition of the MS-cleavable chemical cross-linker DSSO (Fig. 3*A*). Identified cross-links were first filtered to have at least two CSMs per identified cross-link and inter-protein cross-links were visualized as an interaction network (404 cross-links across 98 protein-protein interactions for 60 proteins). The recombinant spike protein interacted with multiple apolipoproteins on HDL (Fig. 3*B* and supplemental Table S4). Interactions identified were between the S2 domain of spike and ApoA1 as well as between the N-terminal domain of spike and ApoC3. ApoD was the only spike-interactor to be cross-linked within the receptor-binding domain (RBD) of the spike (Fig. 3*C*). The cross-link with ApoD occurred at lysine residue K417 within the spike-RBD. This residue is a key salt-bridge-forming residue in the interaction between the spike and its receptor angiotensin-converting enzyme 2 (ACE2) (36) (Fig. 3*C*). K417 within the spike-RBD is also a residue commonly mutated in variants of SARS-CoV-2, including the Omicron variant (K417N) (37).

### Validation of the Interaction Between ApoD and SARS-CoV-2 Spike

**Fig. 4:**
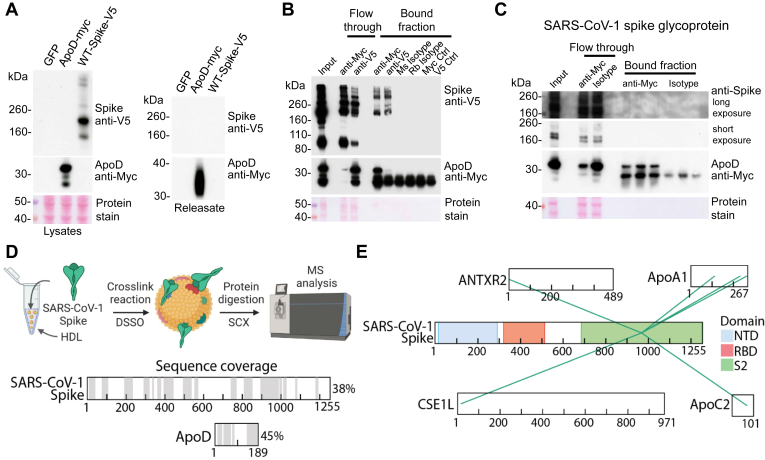
**Specificity of ApoD and SARS-CoV-2 spike protein interaction.***A*, HEK293T cells were transfected with plasmids encoding GFP, ApoD-myc, or spike-V5 prior to Western blot analysis of cell lysates and releasates confirming expression. *B*, co-immunoprecipitations of both ApoD-myc and spike-V5 were conducted upon HEK293T cell lysates co-overexpressing both ApoD-myc and spike-V5. Flow-throughs represent depleted lysates, and bound-fractions are proteins eluted from protein G beads. Myc Ctrl represents an anti-myc immunoprecipitation conducted upon cell lysates singularly expressing spike-V5. V5 Ctrl represents an anti-V5 immunoprecipitation conducted upon cell lysates singularly expressing ApoD-myc. *C*, HEK293T cells were co-transfected in triplicate with plasmids encoding the SARS-CoV-1 spike protein and ApoD-myc prior to immunoprecipitation using anti-myc antibodies, and pull downs were confirmed by Western blot. *D*, XL-MS with SARS-CoV-1 spike protein and the identified peptide coverage for spike and ApoD is schematically visualized. *E*, protein-protein interactions identified by XL-MS with SARS-CoV-1 spike with only one CSM are separately visualised. Spike structural domains are coloured; NTD: *blue*, RBD: *red* and S2: *green*. XL-MS data visualisation was conducted in xiview.org. All protein abbreviations are gene names as determined by Uniprot.

To further validate the interaction between the SARS-CoV-2 spike glycoprotein and ApoD, HEK293T cells were transfected with plasmids encoding the Wuhan (WT) SARS-CoV-2 spike protein (V5-tagged) and ApoD (myc-tagged). Expression of both spike and ApoD was confirmed in cell lysates, while only ApoD was observed in the cell culture supernatants due to the spike plasmid not encoding a secretion sequence (Fig. 4*A*). Utilizing both anti-V5 and anti-myc immunoprecipitation revealed an interaction between spike and ApoD, whereby the pulldown of spike co-immunoprecipitated ApoD and the pulldown of ApoD co-immunoprecipitated spike (Fig. 4*B*). These data from cell lysates highlight the potential of ApoD and SARS-CoV-2 spike protein to interact intracellularly. The specificity of both the anti-V5 and anti-myc antibodies used were confirmed in singularly expressing lysates as well as through the use of an anti-ApoD antibody which also co-immunoprecipitated spike (Fig. 4*B* and supplemental Fig. S2).

### Specificity of Interactions Compared to SARS-CoV-1 Spike With HDL

Next, we determined the specificity of the interaction between ApoD and SARS-CoV-2 spike glycoprotein. SARS-CoV-1 spike lacks K417 found in the SARS-CoV-2 spike protein that we observed to cross-link with ApoD (Fig. 3*C*). We, therefore, investigated whether ApoD has the propensity to bind the SARS-CoV-1 spike glycoprotein. Utilizing overexpression of both ApoD and SARS-CoV-1 spike (untagged), we were unable to detect a notable interaction between ApoD and SARS-CoV-1 spike when using an anti-myc immunoprecipitation, as determined by Western blot (Fig. 4*C*). Thus, ApoD shows preferential binding to the SARS-CoV-2 spike glycoprotein.

To further exclude the propensity of the SARS-CoV-1 spike to interact with ApoD we conducted another XL-MS experiment. Again, HDL was supplemented with recombinant SARS-CoV-1 spike protein prior to the addition of DSSO and further sample processing for XL-MS analysis (Fig. 4*D*). The identified peptide sequence coverage identified for SARS-CoV-1 spike and ApoD is represented, highlighting a broad coverage across both proteins, particularly within the RBD of the spike (Fig. 4*D*). Consistent with the above immunoprecipitation data, we were unable to detect an interaction between SARS-CoV-1 spike and ApoD. Even when spike interactors with only one CSM per cross-link were explored, no interaction was observed between spike and ApoD, although interactions were seen between spike and ApoA1, ApoC2, ANTXR cell adhesion molecule 2 (ANTXR2), and exportin-2 (CSE1L), all within the S2 domain of spike (Fig. 4*E* and supplemental Table S5). The interprotein cross-links between SARS-CoV-1 spike and ApoA1 highlight a commonality between both spike proteins to interact with the core apolipoprotein of HDL.

### Immunoprecipitation-MS Identifies PGRMC1 as SARS-CoV-2 Spike Binder

**Fig. 5:**
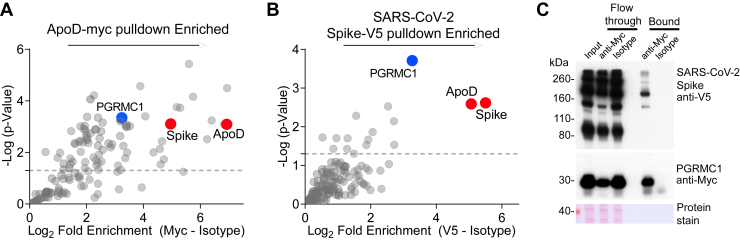
**Immunoprecipitation-MS uncovers interactions with PGRMC1.***A* and *B*, immunoprecipitation-MS was conducted to confirm the interaction between ApoD and spike, upon anti-myc (n = 3), anti-V5 (n = 4) and isotype control (n = 6) eluted fractions, data is represented as volcano plots highlighting fold enrichments over isotype control samples. Significance was determined by Welch’s *t* test. *C*, HEK293T cells were transfected with plasmids encoding PGRMC1-myc and spike-V5 prior to immunoprecipitation using an anti-myc tag antibody. The pulldown of both PGRMC1 and spike was confirmed by Western blot.

The immunoprecipitation samples described in Figure 4 were further analyzed by MS. The co-immunoprecipitation of both ApoD-myc and spike-V5 was confirmed by MS as shown by enrichment of both proteins when compared to isotype controls (Fig. 5, *A* and *B*; supplemental Table S6). Upon further interrogation of the immunoprecipitation-MS datasets, it was observed that membrane-associated progesterone receptor component 1 (PGRMC1) was common in both the pulldowns of ApoD and SARS-CoV-2 spike (Fig. 5, *A* and *B*). Of note, ApoD is a binder and carrier of progesterone (32, 38). Second, PGRMC1 is a known interactor of both the low-density lipoprotein receptor and the very-low density lipoprotein receptor, contributing to lipoprotein uptake and adiposity (39). Due to the novelty of the interaction between the SARS-CoV-2 spike and PGRMC1, we also transfected cells with both PGRMC1-myc and spike-V5 prior to the immunoprecipitation of PGRMC1. SARS-CoV-2 spike was confirmed to interact with PGRMC1, as shown by the enrichment of the SARS-CoV-2 spike in the anti-myc bound fraction compared to isotype control (Fig. 5*C*).

### XL-MS on Recombinant ApoD and SARS-CoV-2 Spike

**Fig. 6:**
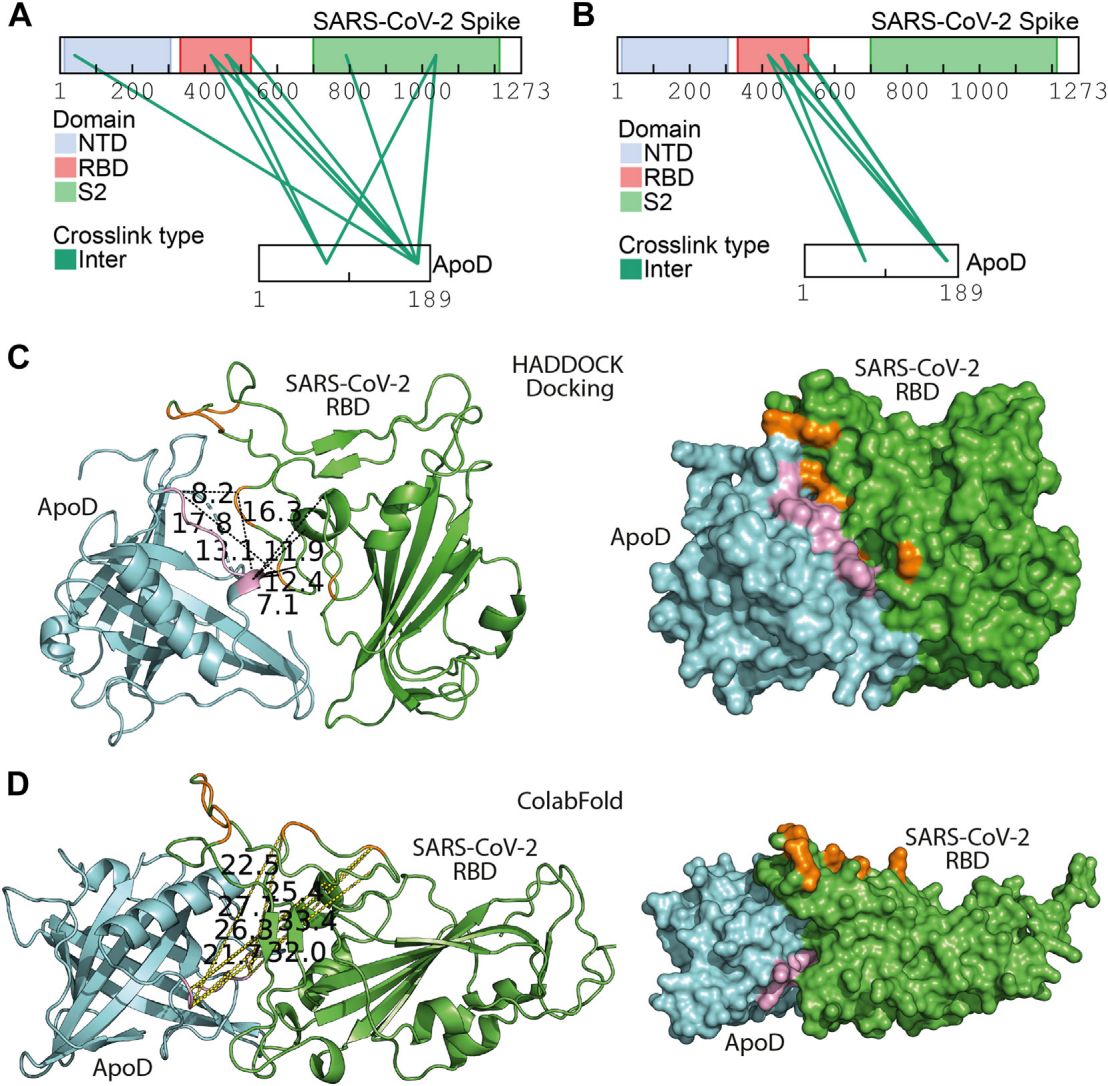
**XLMS of recombinant proteins combined with structural modeling.***A*, an equimolar ratio of full-length SARS-CoV-2 spike and ApoD was mixed prior to crosslinking with DSSO. Identified crosslinks are shown, highlighting a dense crosslinked region between the RBD of spike and the C-terminus of ApoD. *B*, the same experiment was conducted but this time using recombinant spike-RBD, again implicating the C-terminal region of ApoD in this interaction. Spike structural domains are colored; NTD: *blue*, RBD: *red*, and S2: *green*. Publicly available structures of ApoD (pdb.2HZR, *cyan*) and the spike-RBD (pdb.6M0J, *green*) were used to model the interaction between the two proteins. *C*, HADDOCK-based structural modeling was then conducted using identified crosslinks and the likely core amino acid residues (ApoD: *pink*, spike-RBD: *orange*) required for the interaction as identified by DisVis. The top-scoring model conforming to all crosslink distance constraints was visualized in PyMOL and crosslink distances are given in Å. The interaction model was also visualized using surface representation highlighting contacts between key amino acid residues. XL-MS data visualization was conducted in xiview.org. *D*, AlphaFold2 was also used to determine a model between ApoD and spike-RBD. The resulting top-scoring modeling was visualized as described above. Mapped DSSO obtained crosslinks: RBD K417–K155 ApoD, RBD K458–K156 ApoD, RBD K462–K156 ApoD. Mapped DMTMM obtained crosslinks: RBD K458–D161 ApoD, RBD D420–K155 ApoD, RBD E465–K165 ApoD, RBD K462–D161 ApoD.

Due to the lack of cross-link density between ApoD and the SARS-CoV-2 spike-RBD, we next utilized XL-MS with an equimolar mixture of recombinant full-length SARS-CoV-2 spike and recombinant ApoD. Using DSSO, we identified multiple inter-protein cross-links between ApoD and spike (Fig. 6*A* and supplemental Table S7). Consistent with the ApoD-RBD cross-link identified upon HDL, the greatest density of cross-links was observed within the RBD of the spike and the C-terminus of ApoD (Fig. 6*A*). The cross-links between the spike-RBD and ApoD were replicated upon the XL-MS analysis of recombinant spike-RBD and recombinant ApoD (Fig. 6*B* and supplemental Table S8).

So far, we have used the lysine–lysine reactive cross-linker DSSO; however, many cross-linkers exist with differing hetero-bifunctional reactive groups to enable the cross-linking of other amino acid residues. One such example is DMTMM, which when used alone can lead to the cross-linking of lysine residues with acidic amino acids (20). Upon repeating the above experiment with DMTMM rather than DSSO, it was observed that ApoD could interact within the S2 domain of spike (supplemental Fig. S3, *A* and *B*; supplemental Tables S9 and S10). Only a few cross-links between ApoD and the S2 domain of the spike were observed upon the use of DSSO (Fig. 6*A*).

### Computational Modeling Supports Interaction Within the RBD

Next, we modeled the cross-link information gained from the recombinant protein experiments utilizing both DSSO and DMTMM upon the known structures of both ApoD (pdb.2HZR) and the spike-RBD (pdb.6M0J). Using the DisVis software and the cross-links identified between the C-terminus of ApoD and the spike-RBD the interaction space was determined (26, 27, 29). The interaction space between the two proteins is obtained using the known distance constraints of DSSO and the lysine side chains (<30 Å) and DMTMM (<20 Å), and output of suspected active amino acid residues that play a core role within the binding interface is provided (pink and orange residues, Fig. 6C). This amino acid interface, alongside the identified cross-links, were used as an input for the high ambiguity driven protein–protein docking (HADDOCK) platform (15, 28). Cross-links can then be mapped onto predicted interaction models to determine the violation of distance constraints. Models obtained from HADDOCK were individually interrogated to determine the satisfaction of cross-link inputs, revealing an interaction between the C-terminus of ApoD and the same region within the spike-RBD that is known to interact with ACE2 (Fig. 6*C* and supplemental Table S11). Upon visualization using surface representation, the putative interface residues as determined by DisVis were all forming contacts, further supporting interaction between both ApoD and the RBD of the spike (Fig. 6*C*). Following the same workflow, we next modeled the cross-links between ApoD and the S2 domain of spike. In contrast to the results for the spike-RBD, HADDOCK-based prediction modeling of ApoD (pdb.2HZR) and S2-spike (pdb.7DF3) was unable to produce interaction models that satisfied the distance constraints of DMTMM (Supplemental Fig. S3, *C* and *D*).

To complement the docking approach utilized by HADDOCK with experimentally determined cross-links we sought to examine whether AlphaFold2 (30) was capable of predicting the interaction interface between the RBD and ApoD that would satisfy cross-links observed. The top five ranked models from AlphaFold2 showed a large degree of similarity (supplemental Fig. S4 and supplemental Table S12). The top-scoring model is represented in Figure 6*D*. Although on average cross-link distances were greater than that observed from HADDOCK, most cross-links were below 30 Å. The increased distance was driven by the alteration in the placement of the flexible loop of the RBD. Importantly, the conformation adopted by the RBD obtained from AlphaFold2 closely matches that found on spike trimers with an RBD in an up position, suggesting ApoD accessibility to the required RBD residues (Fig. 6*D*).

### No Effects on Viral Replication or Infectivity

**Fig. 7:**
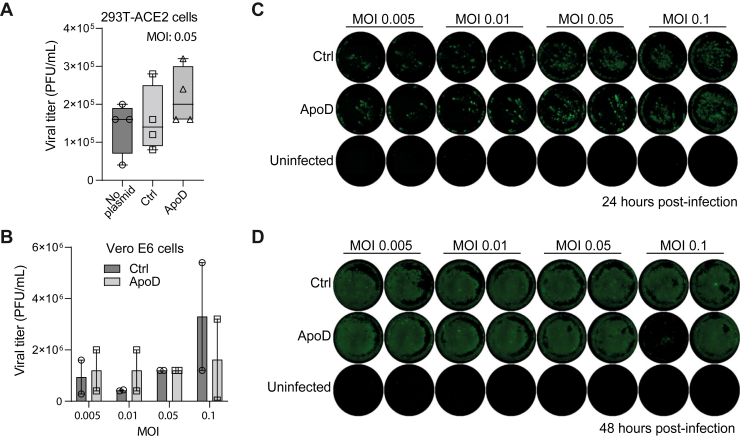
**ApoD and SARS-CoV-2 replication or infection.***A*, the replicative ability of SARS-CoV-2 (multiplicity of infection: MOI 0.05) was determined in HEK293T-ACE2 cells overexpressing ApoD or control empty vector, viral titers were determined 48 h post-infection (n = 4). *B*, the replicative ability of SARS-CoV-2 was further determined in Vero E6 cells overexpressing ApoD or empty vector at increasing concentrations of SARS-CoV-2 (MOI 0.005–0.1, n = 2 per MOI), viral titres were measured 48 h post-infection (plaque forming units, PFU/ml). *C* and *D*, SARS-CoV-2 infectivity was determined in Vero E6 cells overexpressing ApoD or empty vector. Cells were infected with SARS-CoV-2 at the indicated MOIs, and N protein immunofluorescence was measured 24 and 48 h post-infection.

Finally, based on the observed interaction between ApoD and the RBD region of SARS-CoV-2 spike that is responsible for binding the host receptor ACE2 (Fig. 6), we sought to determine whether ApoD was capable of modulating SARS-CoV-2 functionality *in vitro*. Both HEK293T-ACE2 (Fig. 7*A*) and Vero E6 cells (Fig. 7*B*) were first used to determine the influence of ectopically expressed ApoD upon SARS-CoV-2 replication. In both cell types, overexpression of ApoD had no effect upon viral replication (48 h post infection) when compared with controls (Fig. 7, *A* and *B*). Finally, Vero E6 cells were transfected with either empty vector (Ctrl) or ApoD prior to infection with increasing concentrations of SARS-CoV-2 (MOI 0.005, 0.01, 0.05 and 0.1), with the extent of infection being determined by N protein immunofluorescence 24 and 48 h post-infection. No difference in SARS-CoV-2 infection was observable in cells overexpressing ApoD compared with control at either 24 (Fig. 7*C*) or 48 h (Fig. 7*D*) post-infection. Thus, soluble ApoD, devoid of lipoproteins, does not affect viral replication or infectivity across the viral concentrations used.

## Discussion

Utilizing XL-MS, immunoprecipitation-MS, and data-driven structural modeling, we explored SARS-CoV-2 spike protein interactions with HDL. XL-MS enabled the identification of multiple modalities in which spike may interact with HDL, including an interaction with ApoD which was confirmed by immunoprecipitation. The application of XL-MS on plasma from COVID-19 patients revealed acute-phase HDL remodeling, dominated by SAA that also interacts with ApoD. While the interactions of recombinant ApoD were mapped to the RBD of SARS-CoV-2 spike protein, cellular overexpression of ApoD did not influence SARS-CoV-2 replication or infectivity.

Multiple proteomics studies have associated plasma ApoD levels with both COVID-19 severity and mortality (1, 2, 3, 4). These observational findings do not necessarily indicate causality. Our previous study using immunoprecipitation MS in plasma from COVID-19 patients (1), supported a potential interaction of ApoD with the SARS-CoV-2 spike protein. ApoD is highly expressed in the lung (40) and has been shown to be increased in patients with chronic obstructive pulmonary disorder (41) as well as in the lungs of mice during SARS-CoV infection (42). Recently, ApoD expression was also reported to be increased in SARS-CoV-2 infected cardiac tissue (43). Of note, ApoD expression in the brain has been previously shown to be protective against human coronavirus OC43-induced encephalitis; however, the ability of ApoD to directly interact with the virus was not determined (44).

While initial interaction between HDL and SARS-CoV-2 would most likely occur in the lung, multiple organs are now understood to be a direct target of infection, including the liver (45) and intestine (46, 47), critical sites of lipoprotein biogenesis and metabolism. However, the mode of viral dissemination to these secondary target organs remains unclear. SARS-CoV-2 RNA can be detected within the circulation of patients with COVID-19 but is thought not to be associated with infectious virions (1, 48). It has been proposed that the intestine, an active site of viral shedding, may subsequently enable hepatocyte infection (45). It should be noted that the intestine is responsible for approximately 30% of circulating HDL particles (49) while also expressing SR-BI (50) and ApoD (40) to a large degree. Thus, interactions between SARS-CoV-2 and lipoproteins may be occurring within the intestine during biogenesis, enabling viral transit to the liver for further dissemination.

In our previous work, we identified galectin-3 binding protein (LGALS3BP), as well as ApoD, as interactors of the spike through immunoprecipitation-MS of plasma of patients with COVID-19 supplemented with recombinant spike protein (1). Although overexpression of LGALS3BP was capable of reducing syncytia formation and pseudoparticle uptake, the use of exogenous LGALS3BP had no effect (1). In line with this work, it has also recently been shown that exogenous LGALS3BP failed to exert antiviral effects (51). Of interest, LGALS3BP is also a known interactor of HDL, with a cross-link being observed with ApoA1 in our dataset (52). The apparent lack of effect of ApoD upon SARS-CoV-2 infection highlights the difficulty in the identification of functional soluble interaction partners of spike that may rely heavily on high local concentrations.

As we explored, ApoD is primarily carried by HDL in the circulation, where it can form a disulfide bridge with ApoA2 while also sampling dimer and tetramer conformations (53). While we were able to confirm an interaction between spike and ApoD upon HDL, overexpression of soluble ApoD in HEK293T and Vero E6 cells did not affect viral replication or infectivity. However, it has to be noted that overexpression of ApoD fails to capture its lipoprotein association which may be critical with regard to SARS-CoV-2. The immunoprecipitation MS analyses also revealed novel putative binding partners. To the best of our knowledge, an interaction between ApoD and PGRMC1 has not previously been observed. Although spike and PGRMC1 have not previously been identified to interact, phospho-proteomics in Vero E6 cells upon SARS-CoV-2 infection revealed a significant increase in the phosphorylation state of PGRMC1 (S57, S181, *p* = 1.1e-06 and 2.0e-06, respectively) (54). Furthermore, PGRMC1 is highly expressed in the liver, a target of SARS-CoV-2 infection (45).

A limitation of our study was the requirement to use a high concentration of both HDL and spike protein for XL-MS which may lead to non-specific interactions. However, we have previously used this methodology in the context of PCSK9-HDL binding. In this study, recombinant PCSK9 was added to HDL prior to XL-MS. Although we identified eight potential interactors of PCSK9 with HDL proteins, ApoD was not one of these. To further rule out non-specific interactions, we used SARS-CoV-1 spike protein as a control. Another limitation of our study is that HDL interactions were not studied over time. In our previous work, however, apolipoproteins were among the proteins that showed the highest degree of variation in circulating abundance over the time course of COVID-19, highlighting the importance of disease stage in the potential interaction between lipoproteins and SARS-CoV-2 (1).

In summary, our study provides the first example of XL-MS conducted upon isolated HDL and plasma in the context of SARS-CoV-2, providing a repository of novel SARS-CoV-2 spike protein interactions. We uncover potential molecular mechanisms for how SARS viruses may interact with lipoproteins. The failure to observe an interaction between SARS-CoV-1 spike and ApoD poses the interesting hypothesis that evolutionary differences between the SARS and SARS-CoV-2 viruses have resulted in differing abilities to bind HDL. Future studies will need to show whether the ability of lipoproteins to interact with SARS-CoV-2 may influence the ability of the immune system to recognize SARS-CoV-2 or impact viral dissemination. Given the important role of metabolic risk factors such as type 2 diabetes and obesity for adverse outcomes in COVID-19, the potential binding of SARS-CoV-2 to lipoproteins is an important avenue to explore (55).

## Data Availability

Mass spectrometry raw data for XL-MS and immunoprecipitation-MS experiments have been deposited to the ProteomeXchange Consortium (http://proteomecentral.proteomexchange.org) *via* the PRIDE partner repository (56) with the dataset identifier PXD036640 reviewer_pxd036640@ebi.ac.uk. Mass spectrometry raw data for the non-depleted COVID-19 plasma XL-MS has been deposited with the dataset identifier PXD041831 reviewer_pxd041831@ebi.ac.uk. Mass spectrometry raw data for the SARS-CoV-1 spike HDL XL-MS has been deposited with the dataset identifier PXD041438 reviewer_pxd041438@ebi.ac.uk. Proteome Discoverer result files have been separately deposited to the ProteomeXchange Consortium to enable viewing of MSMS spectra (http://proteomecentral.proteomexchange.org) *via* the PRIDE partner repository (56) with the dataset identifier PXD042625.

## Supplemental Data

This article contains supplemental data.

## Conflict of interest

The authors declare no competing interests.

## References

[1] Gutmann C., Takov K., Burnap S.A., Singh B., Ali H., Theofilatos K. (2021). SARS-CoV-2 RNAemia and proteomic trajectories inform prognostication in COVID-19 patients admitted to intensive care. Nat. Commun..

[2] Geyer P.E., Arend F.M., Doll S., Louiset M.-L., Virreira Winter S., Müller-Reif J.B. (2021). High-resolution serum proteome trajectories in COVID-19 reveal patient-specific seroconversion. EMBO Mol. Med..

[3] Demichev V., Tober-Lau P., Lemke O., Nazarenko T., Thibeault C., Whitwell H. (2021). A time-resolved proteomic and prognostic map of COVID-19. Cell Syst..

[4] Overmyer K.A., Shishkova E., Miller I.J., Balnis J., Bernstein M.N., Peters-Clarke T.M. (2021). Large-scale multi-omic analysis of COVID-19 severity. Cell Syst..

[5] Masana L., Correig E., Ibarretxe D., Anoro E., Arroyo J.A., Jericó C. (2021). Low HDL and high triglycerides predict COVID-19 severity. Sci. Rep..

[6] Wang Z., Cryar A., Lemke O., Tober-Lau P., Ludwig D., Helbig E.T. (2022). A multiplex protein panel assay for severity prediction and outcome prognosis in patients with COVID-19: an observational multi-cohort study. EClinicalMedicine.

[7] Chidambaram V., Kumar A., Majella M.G., Seth B., Sivakumar R.K., Voruganti D. (2022). HDL cholesterol levels and susceptibility to COVID-19. EBioMedicine.

[8] Hilser J.R., Han Y., Biswas S., Gukasyan J., Cai Z., Zhu R. (2021). Association of serum HDL-cholesterol and apolipoprotein A1 levels with risk of severe SARS-CoV-2 infection. J. Lipid Res..

[9] Grassi G., Di Caprio G., Fimia G.M., Ippolito G., Tripodi M., Alonzi T. (2016). Hepatitis C virus relies on lipoproteins for its life cycle. World J. Gastroenterol..

[10] Wei C., Wan L., Yan Q., Wang X., Zhang J., Yang X. (2020). HDL-scavenger receptor B type 1 facilitates SARS-CoV-2 entry. Nat. Metab..

[11] Sanders D.W., Jumper C.C., Ackerman P.J., Bracha D., Donlic A., Kim H. (2021). SARS-CoV-2 requires cholesterol for viral entry and pathological syncytia formation. Elife.

[12] Cho K.H., Kim J.R., Lee I.C., Kwon H.J. (2021). Native high-density lipoproteins (HDL) with higher paraoxonase exerts a potent antiviral effect against SARS-CoV-2 (COVID-19), while glycated HDL lost the antiviral activity. Antioxidants (Basel).

[13] Graziadei A., Rappsilber J. (2022). Leveraging crosslinking mass spectrometry in structural and cell biology. Structure.

[14] Wheat A., Yu C., Wang X., Burke A.M., Chemmama I.E., Kaake R.M. (2021). Protein interaction landscapes revealed by advanced in vivo cross-linking–mass spectrometry. Proc. Natl. Acad. Sci. U. S. A..

[15] Dominguez C., Boelens R., Bonvin A.M.J.J. (2003). HADDOCK: a protein−protein docking approach based on biochemical or biophysical information. J. Am. Chem. Soc..

[16] Burnap S.A., Sattler K., Pechlaner R., Duregotti E., Lu R. (2021). PCSK9 activity is potentiated through HDL binding. Circ. Res..

[17] Manthei K.A., Patra D., Wilson C.J., Fawaz M.V., Piersimoni L., Shenkar J.C. (2020). Structural analysis of lecithin:cholesterol acyltransferase bound to high density lipoprotein particles. Commun. Biol..

[18] Gutmann C., Khamina K., Theofilatos K., Diendorfer A.B., Burnap S.A., Nabeebaccus A. (2021). Association of cardiometabolic microRNAs with COVID-19 severity and mortality. Cardiovasc. Res..

[19] Liu F., Rijkers D.T., Post H., Heck A.J. (2015). Proteome-wide profiling of protein assemblies by cross-linking mass spectrometry. Nat. Methods.

[20] Hevler J.F., Zenezeni Chiozzi R., Cabrera-Orefice A., Brandt U., Arnold S., Heck A.J.R. (2021). Molecular characterization of a complex of apoptosis-inducing factor 1 with cytochrome c oxidase of the mitochondrial respiratory chain. Proc. Natl. Acad. Sci. U. S. A..

[21] Liu F., Lössl P., Scheltema R., Viner R., Heck A.J.R. (2017). Optimized fragmentation schemes and data analysis strategies for proteome-wide cross-link identification. Nat. Commun..

[22] Chen Z.-L., Meng J.-M., Cao Y., Yin J.-L., Fang R.-Q., Fan S.-B. (2019). A high-speed search engine pLink 2 with systematic evaluation for proteome-scale identification of cross-linked peptides. Nat. Commun..

[23] Graham M., Combe C., Kolbowski L., Rappsilber J. (2019). xiView: a common platform for the downstream analysis of crosslinking mass spectrometry data. bioRxiv.

[24] Dicks M.D.J., Rose L.M., Russell R.A., Bowman L.A.H., Graham C., Jimenez-Guardeño J.M. (2022). Modular capsid decoration boosts adenovirus vaccine-induced humoral immunity against SARS-CoV-2. Mol. Ther..

[25] Khan H., Winstone H., Jimenez-Guardeño J.M., Graham C., Doores K.J., Goujon C. (2021). TMPRSS2 promotes SARS-CoV-2 evasion from NCOA7-mediated restriction. PLoS Pathog..

[26] van Zundert G.C.P., Bonvin A.M.J.J. (2015). DisVis: quantifying and visualizing accessible interaction space of distance-restrained biomolecular complexes. Bioinformatics.

[27] van Zundert G.C.P., Trellet M., Schaarschmidt J., Kurkcuoglu Z., David M., Verlato M. (2017). The disvis and powerfit web servers: explorative and integrative modeling of biomolecular complexes. J. Mol. Biol.

[28] van Zundert G.C.P., Rodrigues J.P.G.L.M., Trellet M., Schmitz C., Kastritis P.L., Karaca E. (2016). The HADDOCK2.2 web server: user-friendly integrative modeling of biomolecular complexes. J. Mol. Biol..

[29] Honorato R.V., Koukos P.I., Jiménez-García B., Tsaregorodtsev A., Verlato M., Giachetti A. (2021). Structural biology in the clouds: the WeNMR-EOSC ecosystem. Front. Mol. Biosci..

[30] Mirdita M., Schütze K., Moriwaki Y., Heo L., Ovchinnikov S., Steinegger M. (2022). ColabFold: making protein folding accessible to all. Nat. Methods.

[31] Wilson P.G., Thompson J.C., Shridas P., McNamara P.J., Beer M.C.d., Beer F.C.d. (2018). Serum amyloid a is an exchangeable apolipoprotein. Arterioscler. Thromb. Vasc. Biol..

[32] Eichinger A., Nasreen A., Kim H.J., Skerra A. (2007). structural insight into the dual ligand specificity and mode of high density lipoprotein association of apolipoprotein D. J. Biol. Chem..

[33] McConathy W.J., Alaupovic P. (1976). Studies on the isolation and partial characterization of apolipoprotein D and lipoprotein D of human plasma. Biochemistry.

[34] McConathy W.J., Alaupovic P. (1973). Isolation and partial characterization of apolipoprotein D: a new protein moiety of the human plasma lipoprotein system. FEBS Lett..

[35] Braesch-Andersen S., Beckman L., Paulie S., Kumagai-Braesch M. (2014). ApoD mediates binding of HDL to LDL and to growing T24 carcinoma. PLoS One.

[36] Lan J., Ge J., Yu J., Shan S., Zhou H., Fan S. (2020). Structure of the SARS-CoV-2 spike receptor-binding domain bound to the ACE2 receptor. Nature.

[37] McCallum M., Czudnochowski N., Rosen L.E., Zepeda S.K., Bowen J.E., Walls A.C. (2022). Structural basis of SARS-CoV-2 Omicron immune evasion and receptor engagement. Science.

[38] Kielkopf C.S., Ghosh M., Anand G.S., Brown S.H.J. (2019). HDX-MS reveals orthosteric and allosteric changes in apolipoprotein-D structural dynamics upon binding of progesterone. Protein Sci..

[39] Furuhata R., Kabe Y., Kanai A., Sugiura Y., Tsugawa H., Sugiyama E. (2020). Progesterone receptor membrane associated component 1 enhances obesity progression in mice by facilitating lipid accumulation in adipocytes. Commun. Biol..

[40] Perdomo G., Dong H.H. (2008). Apolipoprotein D in lipid metabolism and its functional implication in atherosclerosis and aging. Aging.

[41] Morrow J.D., Chase R.P., Parker M.M., Glass K., Seo M., Divo M. (2019). RNA-sequencing across three matched tissues reveals shared and tissue-specific gene expression and pathway signatures of COPD. Respir. Res..

[42] Kim J., Zhang J., Cha Y., Kolitz S., Funt J. (2020). Advanced bioinformatics rapidly identifies existing therapeutics for patients with coronavirus disease-2019 (COVID-19). J. Transl. Med..

[43] Bräuninger H., Stoffers B., Fitzek A.D.E., Meißner K., Aleshcheva G., Schweizer M. (2021). Cardiac SARS-CoV-2 infection is associated with pro-inflammatory transcriptomic alterations within the heart. Cardiovasc. Res..

[44] Do Carmo S., Jacomy H., Talbot P.J., Rassart E. (2008). Neuroprotective effect of apolipoprotein D against human coronavirus OC43-induced encephalitis in mice. J. Neurosci..

[45] Wanner N., Andrieux G., Badia-i-Mompel P., Edler C., Pfefferle S., Lindenmeyer M.T. (2022). Molecular consequences of SARS-CoV-2 liver tropism. Nat. Metab..

[46] Qian Q., Fan L., Liu W., Li J., Yue J., Wang M. (2020). Direct evidence of active SARS-CoV-2 replication in the intestine. Clin. Infect. Dis..

[47] Lamers M.M., Beumer J., van der Vaart J., Knoops K., Puschhof J., Breugem T.I. (2020). SARS-CoV-2 productively infects human gut enterocytes. Science.

[48] Andersson M.I., Arancibia-Carcamo C.V., Auckland K., Baillie J.K., Barnes E., Beneke T. (2020). SARS-CoV-2 RNA detected in blood products from patients with COVID-19 is not associated with infectious virus. Wellcome Open Res..

[49] Brunham L.R., Kruit J.K., Iqbal J., Fievet C., Timmins J.M., Pape T.D. (2006). Intestinal ABCA1 directly contributes to HDL biogenesis in vivo. J. Clin. Invest..

[50] Altmann S.W., Davis H.R., Yao X., Laverty M., Compton D.S., Zhu L.J. (2002). The identification of intestinal scavenger receptor class B, type I (SR-BI) by expression cloning and its role in cholesterol absorption. Biochim. Biophys. Acta.

[51] de Jarcy L.B., Akbil B., Leyens J., Postmus D., Harnisch G., Jansen J. (2022). 90K/LGALS3BP expression is upregulated in COVID-19 but does not restrict SARS-CoV-2 infection. medRxiv.

[52] Furtado J.D., Yamamoto R., Melchior J.T., Andraski A.B., Gamez-Guerrero M., Mulcahy P. (2018). Distinct proteomic signatures in 16 HDL (high-density lipoprotein) subspecies. Arterioscler. Thromb. Vasc. Biol..

[53] Yang C.Y., Gu Z.W., Blanco-Vaca F., Gaskell S.J., Yang M., Massey J.B. (1994). Structure of human apolipoprotein D: locations of the intermolecular and intramolecular disulfide links. Biochemistry.

[54] Bouhaddou M., Memon D., Meyer B., White K.M., Rezelj V.V., Marrero M.C. (2020). The global phosphorylation landscape of SARS-CoV-2 infection. Cell.

[55] Bechmann N., Barthel A., Schedl A., Herzig S., Varga Z., Gebhard C. (2022). Sexual dimorphism in COVID-19: potential clinical and public health implications. Lancet Diabetes Endocrinol..

[56] Vizcaíno J.A., Deutsch E.W., Wang R., Csordas A., Reisinger F., Ríos D. (2014). ProteomeXchange provides globally coordinated proteomics data submission and dissemination. Nat. Biotechnol..

