# Supplementary material for: Cross-Linking Mass Spectrometry Uncovers Interactions Between High-Density Lipoproteins and the SARS-CoV-2 Spike Glycoprotein
Source: Mol Cell Proteomics. 2023 Jun 19;22(8):100600. doi: 10.1016/j.mcpro.2023.100600 (PMC10279469; doi:10.1016/j.mcpro.2023.100600)
Supplement: Supplemental Figures S1–S4 [file mmc2.pdf]

supplemental Fig. 1

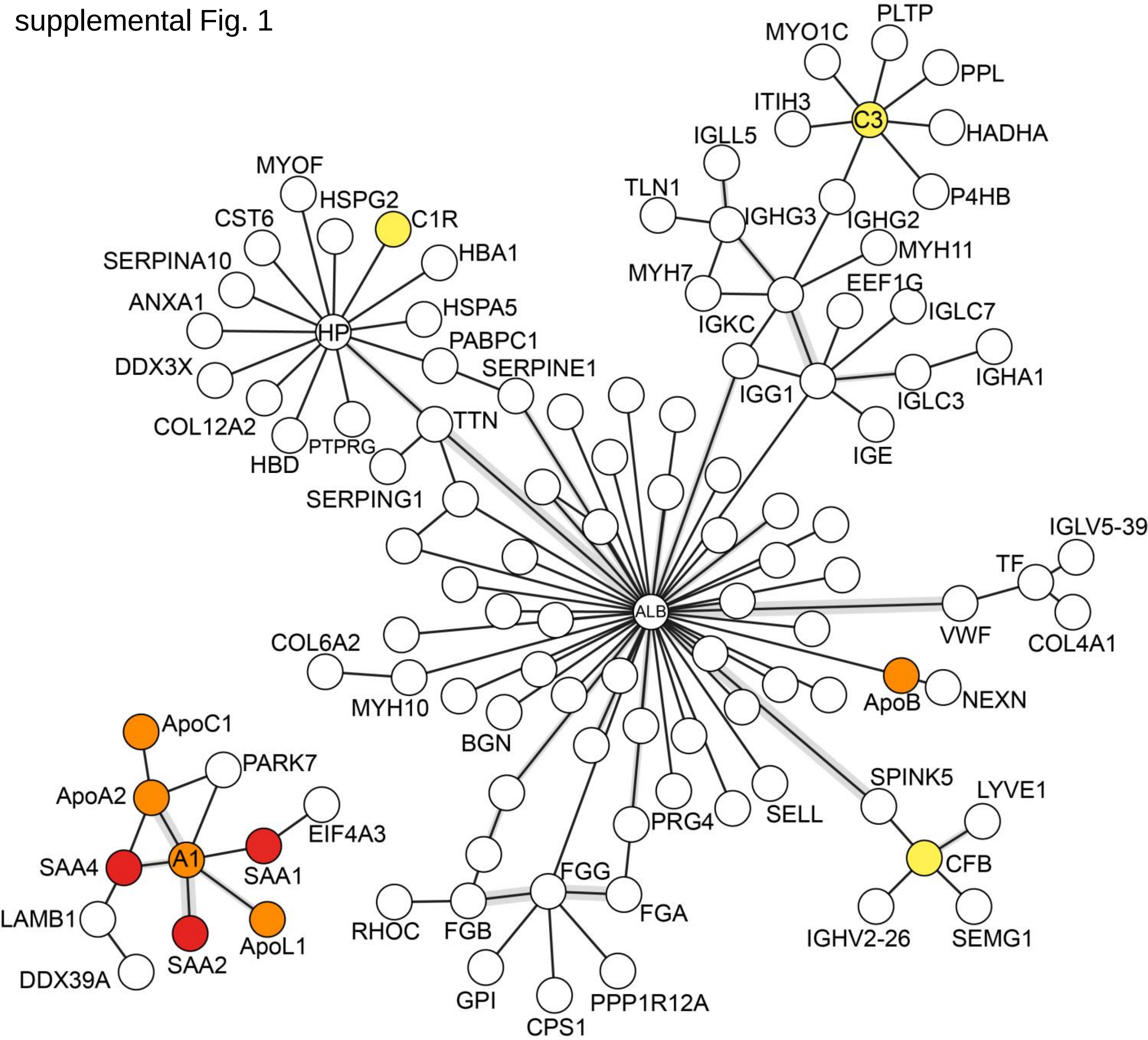

XL-MS upon non-depleted plasma derived from patients with COVID-19. Plasma isolated from patients in intensive care with confirmed COVID-19 (n=6) was pooled and crosslinked using DSSO. The subsequent inter-protein crosslinks identified by XL-MS are represented as an interaction network, 173 crosslinks across 117 proteins. Crosslinks had a minimum of 2 associated crosslink spectral matches (CSMs). Apolipoproteins are in orange, serum amyloid proteins (SAA) are in red and complement factors are in yellow. XL-MS data visualisation was conducted in xiview.org. All protein abbreviations are gene names as determined by Uniprot.

supplemental Fig. 2

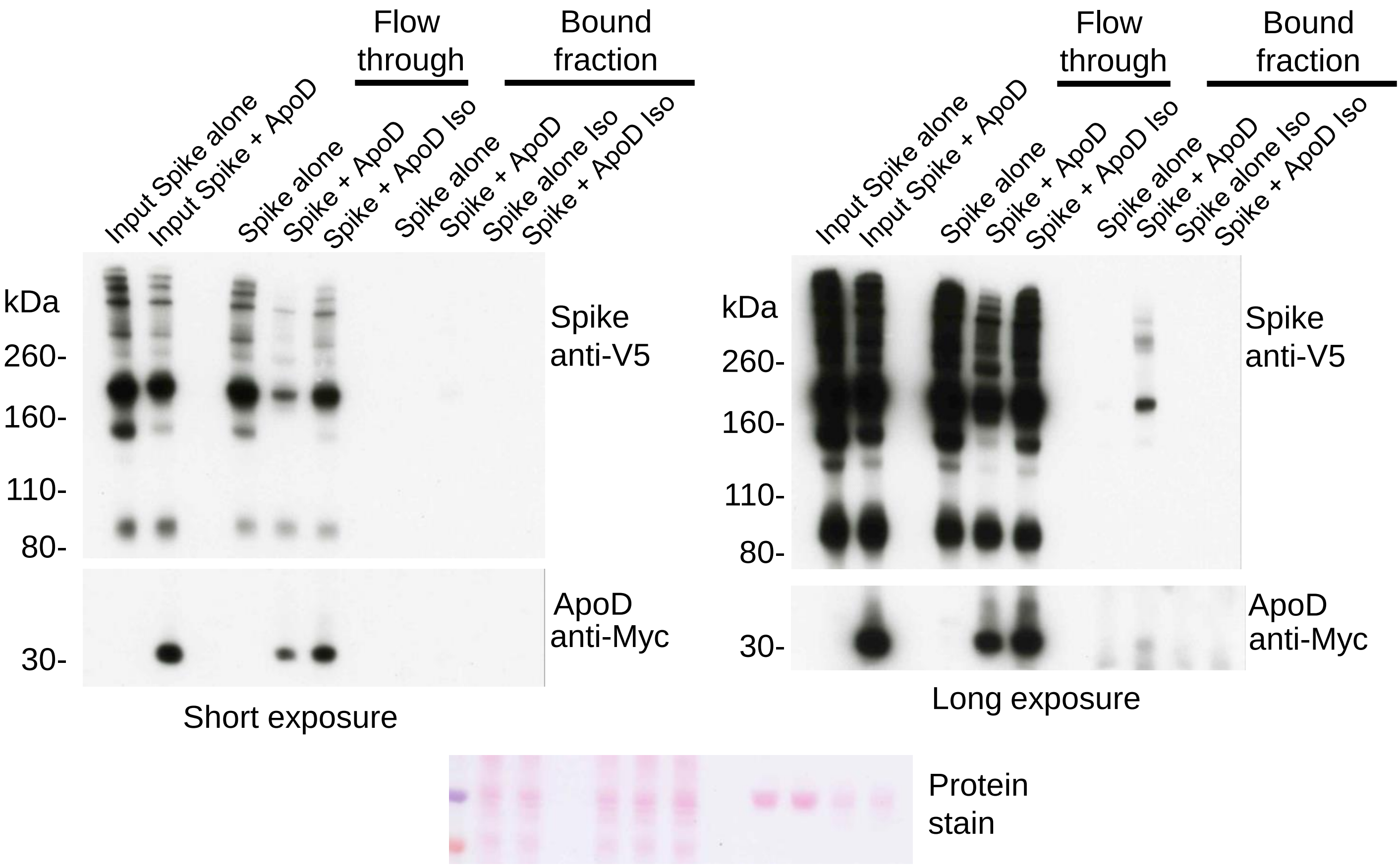

In vitro immunoprecipitation of ApoD and spike. HEK293T cells were transfected with plasmids encoding ApoD-myc and spike-V5 prior to immunoprecipitation using an antibody targeting ApoD. The co-immunoprecipitation of spike was confirmed by Western blot analysis and two exposures are shown. Cells expressing spike-V5 alone were used to control for the specificity of the anti-ApoD antibody.

supplemental Fig. 3

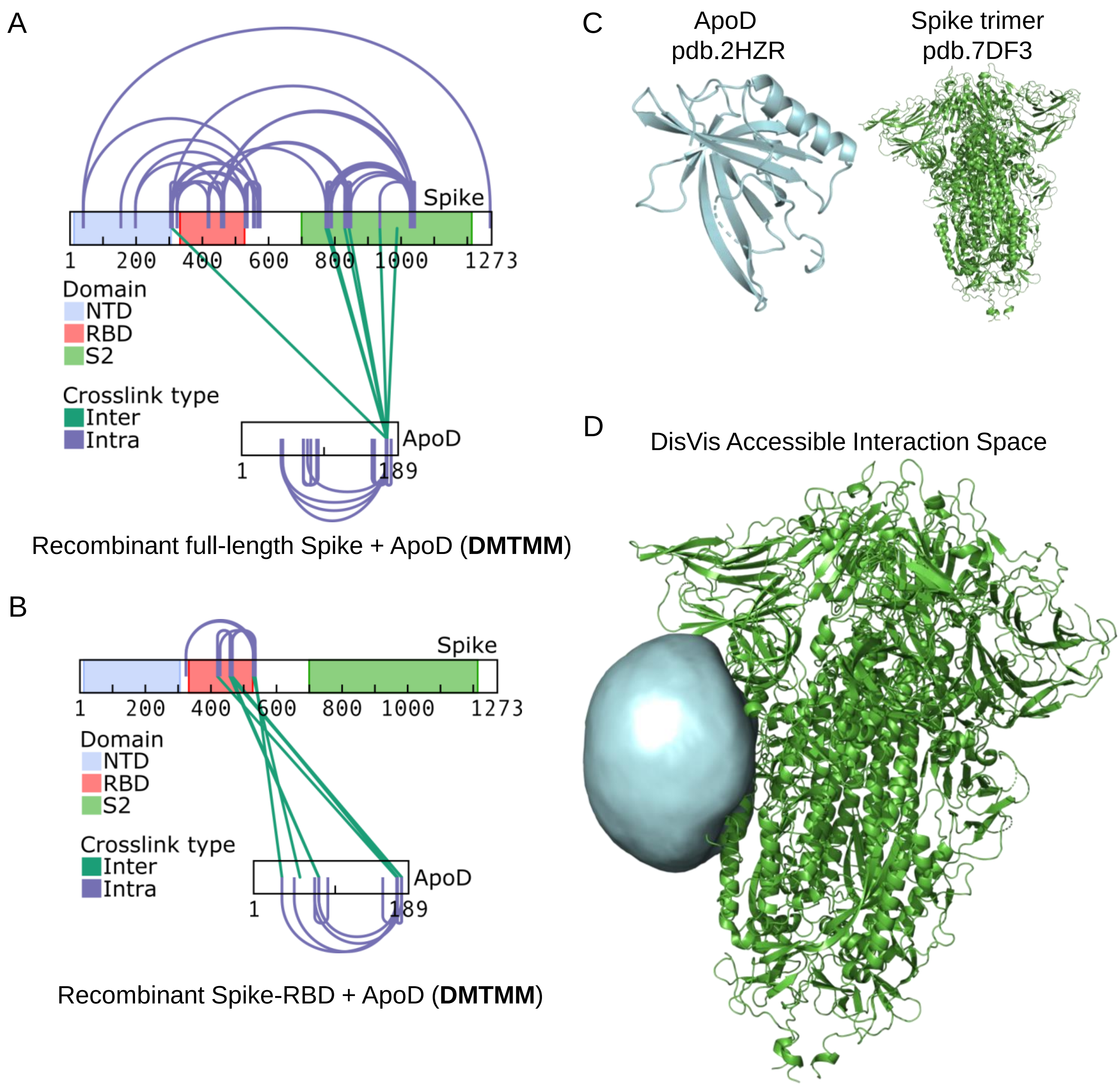

DMTMM XLMS and data-driven structural modelling of the interaction between spike and ApoD. An equimolar ratio of full-length SARS-CoV-2 spike (**A**) or spike-RBD (**B**) and ApoD was mixed prior to the crosslinking with DMTMM. Identified crosslinks are shown, highlighting crosslinked regions between the RBD or S2 domain of spike and the c-terminus of ApoD. **C,** Publicly available structures of ApoD (pdb.2HZR, cyan) and the spike trimer (pdb.7DF3, green) were used to model the interaction between the two proteins. **D,** The DisVis software was used to obtain an interaction interface between spike-S2 and ApoD utilising crosslinks identified. HADDOCK-based modelling failed to produce interaction models that satisfied crosslink distance constraints.

supplemental Fig. 4

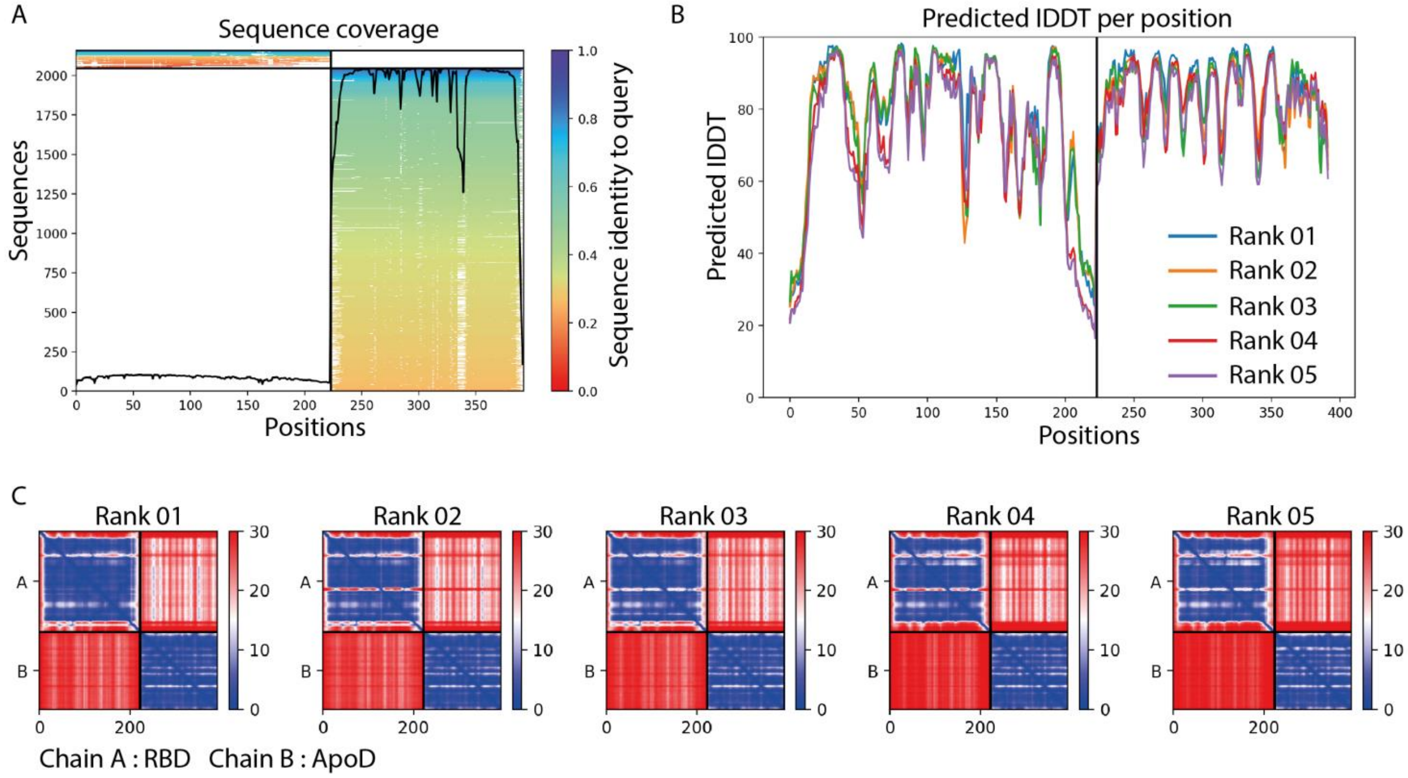

AlphaFold2 modelling and the interaction between ApoD and SARS-CoV-2 RBD. A, the identified sequence coverage across RBD and ApoD is shown. **B**, the per residue estimate of confidence (pLLDT) across the 5 top ranked models is graphically represented. **C**, the predicted alignment error (PAE) for each of the top 5 ranked models are shown as heatmaps.
